# Surveillance radiologic imaging after treatment of oropharyngeal cancer: a review

**DOI:** 10.1186/s12957-015-0481-1

**Published:** 2015-03-07

**Authors:** Steven J Wang

**Affiliations:** Department of Otolaryngology-Head and Neck Surgery, University of California, 2233 Post St, 3rd Floor, San Francisco, CA 94115 USA

**Keywords:** Head and neck cancer, Radiologic imaging, Oropharynx cancer, MRI, PET/CT, Ultrasound

## Abstract

The increasing proportion of human papilloma virus-related oropharynx cancers has led to improved success in the treatment of this disease. However, the current low recurrence rate after treatment of oropharyngeal cancer highlights the continued need for, as well as the challenges of, designing an effective follow-up surveillance program. There are frequently multiple modalities used in the treatment of oropharyngeal cancer, resulting in short- and long-term tissue changes to the head and neck that challenge clinical distinction of recurrence *versus* treatment-related changes. The oropharynx subsite is characterized by complex anatomy not always accessible to physical exam, making radiologic imaging a potentially useful supplement for effective follow-up assessment. In this manuscript, the literature regarding the type of radiologic imaging modality and the frequency of obtaining imaging studies in the surveillance follow-up after treatment of oropharyngeal cancer is reviewed. While ultrasound and MRI have useful characteristics that deserve further study, PET/CT appears to have the best sensitivity and specificity for imaging surveillance follow-up of head and neck cancers including oropharyngeal cancer. A negative PET/CT is particularly useful as a predictor of prognosis and can guide the clinician as to when to stop obtaining additional imaging studies in the absence of clinical signs of recurrence. However, there is scant evidence that imaging surveillance can improve survival outcomes. Suggestions to guide future imaging surveillance research studies are provided.

## Review

Treatment of oropharyngeal cancer includes the frequent use of organ-preservation chemoradiation protocols. Reports in the literature have shown high success with this approach, with advanced stage III/IV oropharyngeal cancers having >80% 2- and 5-year disease-free survival rates [1,2]. More recently, primary surgery with emphasis on transoral approaches for early T-stage oropharyngeal cancer has become more common, with similarly favorable results [3]. Nonetheless, some oropharyngeal cancer patients do suffer recurrent disease, emphasizing the need for post-treatment surveillance. The exact benefit of a surveillance program, the type of surveillance program, and the interval and duration of a surveillance program remain undefined. Because the oropharynx can be a difficult anatomic location to evaluate, and this evaluation may be further obscured by treatment-related tissue changes, it has been generally considered that physical examination alone is insufficient as a surveillance method for oropharyngeal cancer. Thus, radiologic imaging studies, in addition to the physical exam, have been commonly employed in cancer surveillance for this disease. The purpose of this manuscript is to review the evidence regarding the role of radiologic imaging for surveillance after treatment of oropharyngeal cancer.

The goal of a head and neck cancer surveillance program is to achieve earlier detection of recurrent cancer compared to patient self-identification through frequent, interval clinical assessment. To be beneficial, earlier detection should increase the likelihood of successful salvage therapy. However, there is limited data with regard to the benefits for any head and neck cancer surveillance program. The increased survival of patients with recurrent tumors diagnosed by routine surveillance reported in some studies have been criticized as being due, in part, to lead time bias (that is, early diagnosis falsely appears to prolong survival) [4]. Agrawal *et al*. found that the majority of patients diagnosed with recurrent head and neck cancer had self-identified clinical symptoms or findings prior to routine surveillance office follow-up [5]. In a subsequent study, survival in the recurrent disease setting appeared to be more dependent on variables such as prior early disease stage and recurrence location (local-only rather than regional or distant) than those associated with follow-up surveillance [6]. There is no doubt that some head and neck cancer patients with recurrence achieve improved disease outcome through identification in an earlier subclinical point in time. However, the current state of scientific evidence in the literature is scant on this issue, highlighting the need for more critical assessments of head and neck cancer surveillance recommendations.

The proper assessment of any cancer surveillance program must consider 1) the recurrence rate, 2) the optimal method for surveillance, and 3) whether earlier detection of recurrence leads to increased rates of successful salvage treatment and improved survival. Currently, in North America, patients with oropharynx cancers, whether treated with chemoradiation or with primary surgery +/− adjuvant therapy, have a low recurrence rate compared to historic data [7]. Recently, Garden *et al*. reported a 5-year overall recurrence rate of 18% following IMRT treatment of oropharyngeal cancer [2]. The rise in human papilloma virus (HPV)-related oropharynx cancers in many parts of the world is a key factor in the recently reported improved treatment results. Ang *et al*. found that HPV-positive oropharyngeal cancer patients had up to twofold greater 5-year overall survival and less than half the recurrence rate of HPV-negative oropharyngeal cancer patients of the same stage [8]. Because of the decreased incidence of smoking, the incidence of tobacco-related oropharyngeal cancer in the USA has declined by 50% while HPV-positive oropharyngeal cancer cases have increased by 225% since 1988, with HPV-positive cancers now comprising the majority of oropharyngeal cancers that occur in the USA today [9]. The low recurrence rate associated with these tumors means that any surveillance program to be of meaningful benefit must have high sensitivity, in order to successfully identify the relatively few recurrences, as well as high specificity, to avoid unnecessary additional testing, intervention, and anxiety since the likelihood of actually having a recurrence is low.

A second consideration for a cancer surveillance program is the selection of the method for surveillance. Lacking any serum tumor marker or other diagnostic test for recurrence, most efforts have focused on radiologic imaging as the primary tool for head and neck cancer surveillance. It has been reported that radiologic imaging adds to the sensitivity of physical exam alone [10]. However, there is presently no consensus regarding the optimal type of imaging surveillance and the recommended frequency of any imaging surveillance program. The current National Comprehensive Cancer Network (NCCN) guidelines recommend a baseline cross-sectional imaging study at 4 to 6 months following treatment of oropharynx cancer, but there are no recommendations for subsequent routine follow-up imaging [11]. Nonetheless, frequent and expensive imaging studies are routinely obtained at many head and neck cancer treatment centers. In contrast, other centers take a more individualized approach, with post-treatment imaging limited to when a recurrent tumor is suspected, in order to confirm the presence of such a lesion and to determine its extent. The type of imaging study obtained also varies from center to center and may include CT, MRI, PET/CT, or ultrasound. Most recent studies of head and neck cancer surveillance have focused on PET/CT. Below, the literature on the utility of the various modalities of post-treatment imaging surveillance for head and neck cancer, generally, and oropharynx cancer, specifically, is reviewed.

### Ultrasound for surveillance

Ultrasound as a surveillance tool has the advantage of lower cost and the capability for use in conjunction with the routine clinical visit and exam. A previous report from our institution comparing ultrasound and PET/CT for staging and surveillance of head and neck and thyroid cancer found superior sensitivity (96.8% *vs*. 90.3%), specificity (93.3% *vs*. 20%), positive predictive value (96% *vs*. 70%), and negative predictive value (93% *vs*. 50%) for ultrasound compared to PET/CT [12]. Another similar study comparing ultrasound with PET/CT found that the two techniques had equivalent accuracy for surveillance of head and neck cancer, and the authors concluded that ultrasound could be considered complementary to PET/CT for detecting subclinical regional recurrences after head and neck cancer treatment [13]. On the other hand, the use of ultrasound as a practical tool for head and neck cancer surveillance is still relatively constrained due to the limited number of practitioners who are skilled in head and neck ultrasonography.

### MRI for surveillance

MRI is used for head and neck cancer surveillance in many centers. Compared to CT or PET/CT, MRI can provide superior anatomic delineation, particularly with regard to lesions near the skull base. It may also be the more useful study if one desires to confirm and assess the extent of a suspected recurrence, so that surgical planning can be done. We previously reported results of 43 patients treated for oropharynx cancer who underwent 252 head and neck MRI scans [14]. In this study, two recurrences were identified on MRI in otherwise asymptomatic patients while routine clinical follow-up and physical examination identified an additional two recurrences. Six patients experienced false-positive surveillance scans that resulted in intervention. Salvage treatment was performed in the two patients with recurrences identified solely by MRI, one of whom remains free of disease at follow-up. The overall sensitivity and specificity of the MRI surveillance program was 50% and 83%, respectively, suggesting that MRI may have limited utility as a surveillance method for oropharynx cancers in otherwise asymptomatic patients. The use of diffusion-weighted MRI may increase the sensitivity for persistent or recurrent head and neck cancer. Vandecaveye and colleagues reported diffusion-weighted MRI to have a sensitivity of 94.6%, specificity of 95.9%, and overall accuracy of 95.5% for the detection of clinically suspected persistent or recurrent head and neck cancer [15].

### PET/CT for surveillance

Studies examining the role of PET in head and neck cancer surveillance have found notable benefits in the initial post-treatment period. Krabbe *et al*. compared PET *versus* regular clinical follow-up for oral cavity and oropharyngeal carcinoma [16]. PET was significantly more sensitive than regular follow-up for identification of recurrence. The benefit of PET was highest for the 3- and 6-month post-treatment scan. Kao *et al*. studied 80 head and neck cancer patients who underwent 240 post-treatment scans at 4- to 6-month intervals over an approximately 3-year period [17]. In their study, the sensitivity was 92%, the specificity 82%, the positive predictive value 42%, and the negative predictive value 98% for detecting locoregional recurrences. A negative *versus* positive PET/CT within 6 months of completion of treatment offered a significant prognostic value (3-year overall survival 100% *vs*. 32%, *P* = 0.01). These authors concluded that PET/CT is a sensitive technique for the detection of recurrent head and neck cancer.

Abgral *et al*. examined the benefit of a single 12-month post-treatment PET/CT scan in 91 patients without clinical evidence of head and neck cancer recurrence [18]. Fifty-two PET/CTs were reported negative and 39 reported positive. The sensitivity was 100%, specificity 85%, negative predictive value 100%, and positive predictive value 77%. These authors concluded that there is benefit to doing a PET/CT at 12 months after treatment for asymptomatic patients. Manikantan *et al*. also suggested that a PET/CT at 12 months may be useful, but that further studies are required to confirm this recommendation [19]. These authors also recommended the use of ultrasound +/− fine-needle aspiration biopsy for follow-up of necks that have not had surgery.

A meta-analysis of 27 manuscripts on the utility of PET scans for the post-treatment follow-up of head and neck cancer was done by Isles and colleagues [20]. While this meta-analysis mostly included studies that examined the utility of PET for the *initial* evaluation of chemoradiation treatment response, their analysis showed an overall pooled sensitivity of 94% for the detection of residual or recurrent disease at the primary site, with a sensitivity of 74% for residual or recurrent neck disease. The negative predictive values were 95% for the primary site and 96% for neck disease, whereas the positive predictive values were 75% for the primary site and 49% for the neck.

McDermott *et al*. considered the negative predictive value of surveillance PET/CT in head and neck cancer [21]. That is, does a negative PET/CT reduce the need for further imaging surveillance. Their study involved 512 patients (31% oropharynx, 31% oral cavity, 19% larynx or hypopharynx, 8% unknown primary). They reported that a single negative PET/CT (214 patients) carries a negative predictive value of 91%, which they considered not adequate to defer further radiologic surveillance. However, two consecutive negative PET/CTs within 6 months (114 patients) carry a 98% negative predictive value, which could obviate further radiologic imaging in the absence of clinical signs of recurrence.

In North America and some other areas of the world, HPV-related oropharynx cancers are increasingly prevalent, with a better associated prognosis and response to treatment [8,9]. Zhang *et al*. examined whether HPV status could augment the predictive utility of PET/CT interpretation in the post-treatment setting [22]. These authors found that in HPV-positive patients, a negative first post-treatment PET/CT is highly predictive of disease-free survival but not in HPV-negative patients. In a follow-up study from this same group, 61 oropharynx cancer patients were analyzed of which 50 (82%) tumors were HPV-positive [23]. The negative predictive value of a negative initial post-treatment PET/CT for HPV-positive oropharynx cancer patients was 93% *versus* 85% for the oropharynx cancer group overall. On multivariate analysis, HPV status and negative initial PET/CT were the only significant predictors of recurrence.

It also appears that the technique of PET/CT is relevant in the utility of the imaging surveillance program. In many centers, the CT component of the combined PET study is with a non-contrast, low-definition technique whose intent is for anatomic correlation of the PET findings only. However, Rangaswamy *et al*. observed an improvement in the detection of locoregional recurrence in head and neck malignancies when F-18 FDG PET is combined with high-resolution contrast-enhanced CT [24]. Their study compared PET/low-definition CT *versus* PET/high-resolution contrast-enhanced CT *versus* physical exam with in-office endoscopy. They found that PET/high-resolution contrast-enhanced CT had superior sensitivity and negative predictive value. They further reported that the findings of the positive PET/CT had an impact on treatment for 9 of the 103 patients in the study.

A final consideration of a cancer surveillance program is whether the earlier detection of subclinical, asymptomatic recurrences leads to improved salvage outcomes. While the experience of most head and neck cancer physicians is that earlier detection of recurrence in oropharynx cancer does increase the opportunity for treatment cure, the evidence in the reported literature is less clear. The likelihood of salvage treatment success depends heavily on the site of recurrence, with local and/or regional recurrences more likely to be salvaged than distant recurrences.

Ho *et al*. studied the impact of PET/CT surveillance for detecting head and neck cancer recurrence at 12 and 24 months post-treatment [25]. In a 10-year retrospective analysis of 284 treated head and neck cancer patients (49% oropharynx, 19% oral cavity, 18% nasopharynx, 8% larynx/hypopharynx, 6% unknown primary), these authors reviewed 175 patients with 3- and 12-month scans and 77 with 3-, 12-, and 24-month scans. The PET/CT detection rate of occult recurrence was 9% at 12 months and 4% at 24 months. There was no difference in 3-year disease-free survival and overall survival for PET/CT detected *versus* clinically detected recurrences. These authors concluded that PET/CT offers uncertain benefit to those with initial 3-month negative PET/CT, although it was acknowledged that larger prospective studies are needed to fully answer this question.

Similarly, Dunsky *et al*. suggested that PET/CT could be an effective surveillance tool for early detection of asymptomatic disease in patients treated for head and neck cancer; however, the outcomes of those patients with identified recurrences remained poor [26]. Their study reported the detection of asymptomatic lesions in 24 of 103 (20%) patients; among the 24 identified recurrences, 20 (82%) of these were distant metastases and 4 (18%) were in locoregional sites.

A difficulty in drawing conclusions from the published literature is the inconsistency in the design and analysis of imaging surveillance studies. Patel *et al*. recently carried out a systematic review of the evidence for use of PET/CT in the post-treatment surveillance follow-up setting for head and neck cancers [27]. These authors found only four head and neck studies which they believed met adequate criteria for quality and that included specific data for long-term follow-up imaging surveillance without pooling data with the *initial* post-treatment evaluation. For these four studies, there was a pooled sensitivity of 75% to 100%, specificity of 92% to 95%, positive predictive value of 50% to 90%, and negative predictive value of 100%. These authors concluded that there is a lack of evidence supporting PET/CT for post-treatment surveillance of head and neck cancers beyond its use in the initial post-treatment evaluation. Standardization of methods of imaging surveillance and emphasis on the importance of conducting prospective multi-institutional studies was recommended.

#### Future directions in surveillance radiologic imaging

There are several methods for radiologic imaging which offer the promise of improved detection of cancer recurrence but have not yet found widespread application for oropharyngeal carcinoma [28-32]. The use of integrated PET/MRI for the evaluation of head and neck cancer has been described [28]. PET/MRI has theoretical advantages over PET/CT due to the superior soft-tissue delineation of malignancies with MRI compared to contrast-enhanced CT and the absence of radiation dose with MRI. Drawbacks with whole-body PET/MRI include motion artifact of solid organ evaluation (lung, liver/abdomen) associated with respiration, which is greater with MRI than with CT, and the prolonged imaging time (typically, in excess of 1 h) of PET/MRI which may be difficult for some patients to tolerate.

Other strategies to improve cancer detection with radiologic imaging include the use of different contrast agents or probes to increase the imaging sensitivity for tumors [29-32]. To date, the most common contrast agents for MRI have been gadolinium or iron oxide. Newer MR contrast agents are being developed with molecular specificities that can produce signal changes when these agents bind to cell surface receptors on tumor cells or extracellular matrix components [29]. These novel cancer-specific contrast agents with enhanced magnetic properties offer the potential to increase the sensitivity of the MR imaging technique for tumor identification. Tumor-specific contrast agents provide the clinician with molecular imaging information that can facilitate cancer diagnosis, monitoring of chemotherapeutic drug delivery, and assessment of the overall response to treatment [30,31].

The use of newer molecular probes other than 18F-fluorodeoxyglucose (18F-FDG) for PET imaging has also been reported [32]. 18F-fluoromethyltyrosine (18F-FMT) has lower sensitivity for head and neck cancer compared to 18F-FDG; however, 18F-FMT appears to be more tumor-specific than 18F-FDG as there is no uptake of this tracer with inflammation, a property which could aid in the identification of persistent regional nodal metastatic disease [32]. The specific hypoxia tracer 18F-fluoroazomycin arabinoside (18F-FAZA) has been described as a method for PET identification of head and neck cancers with the property of greater tumor hypoxia, which in the future could help individualize treatment, for instance, by selecting patients for hypoxia sensitizers or special intensified radiation treatment techniques [33]. Other PET tracers are being developed that can provide better characterization of the tumor microenvironment, proliferation potential, and tumor metabolism, which may aid in the early diagnosis of cancer recurrence [32].

A summary of current and future imaging strategies for the detection of recurrent oropharynx carcinoma, with references, is shown in Table 1.

**Table 1:** **Surveillance radiologic imaging after oropharynx cancer treatment**

**Imaging modality**	**Reference (year)**
Ultrasound	Hwang *et al*. (2009) [12]; Wierzbicka *et al*. (2011) [13]: comparison of US *versus* PET/CT for staging and surveillance of head/neck and thyroid cancer
MRI	Kangelaris *et al*. (2010) [14]: MRI for surveillance after chemoradiation treatment of oropharynx cancer
	Vandecaveye *et al*. (2007) [15]: diffusion-weighted MRI for head/neck cancer after chemoradiation
PET	Krabbe *et al*. (2009) [16]; Kao *et al*. (2009) [17]; Abgral *et al*. (2009) [18]; Manikantan *et al*. (2009) [19]: sensitivity of PET/CT for recurrent head and neck cancer
	Isles *et al*. (2008) [20]: systematic review of PET for follow-up of head/neck cancer after radiation
	McDermott *et al*. (2013) [21]: negative predictive value of PET/CT for head/neck surveillance
	Zhang *et al*. (2011) [22]; Koshkareva *et al*. (2014) [23]: PET/CT for surveillance of HPV+ oropharynx cancer
	Ho *et al*. (2013) [25]; Dunsky *et al*. (2013) [26]; Patel *et al*. (2013) [27]: lack of survival benefit for routine PET/CT surveillance program
Future directions in surveillance imaging	Lee *et al*. (2014) [28]: PET/MRI for head/neck cancer
	Bogdanov and Mazzanti (2011) [29]; Yankeelov *et al*. (2011) [30]; Glunde and Bhujwalla (2011) [31]: newer contrast agents and tumor biomarkers for MR imaging
	Differding *et al*. (2015) [32]; Servagi-Vernat *et al*. (2014) [33]: PET imaging biomarkers in head and neck cancer

## Conclusions

In summary, there is no high-level scientific evidence to guide us to the optimal strategy of radiologic imaging surveillance for patients treated for oropharynx cancer. However, based on mostly retrospective and single-institution case series reports as well as considering mainly studies that reported imaging surveillance results across all head and neck cancer tumor subsites, PET/CT appears the most sensitive and specific imaging modality for surveillance follow-up of treated oropharynx cancer. PET/CT scans obtained between 3 and 6 months after completion of treatment and at 12 months post-treatment have notable implications for prognosis. PET/CT has important clinical utility because of a generally high specificity and negative predictive value. If two consecutive negative PET/CT studies are obtained within an interval between 3 and 6 months, the likelihood of future recurrence is extremely low. HPV status may further enhance the predictive reliability of the PET/CT findings. However, additional studies are needed to quantify the clinical outcome benefits of an imaging surveillance program and to determine the most appropriate duration and frequency of obtaining imaging studies in this patient population.
